# High Three-Dimensional Detection Accuracy in Piezoelectric-Based Touch Panel in Interactive Displays by Optimized Artificial Neural Networks

**DOI:** 10.3390/s19040753

**Published:** 2019-02-13

**Authors:** Shuo Gao, Yanning Dai, Vasileios Kitsos, Bo Wan, Xiaolei Qu

**Affiliations:** 1 School of Instrumentation and Optoelectronic Engineering, Beihang University, Beijing 100083, China; shuo_gao@buaa.edu.cn (S.G.); 16171056@buaa.edu.cn (Y.D.); 2 Beijing Advanced Innovation Center for Big Data-Based Precision Medicine, Beihang University, Beijing 100083, China; 3 Electronic & Electrical Engineering Department, University College London, London WC1E 7JE, UK; kitsos.vasileios@gmail.com; 4 Cicada Canada Inc., Toronto, ON l5v1t7, Canada; sean.b.wan@gmail.com

**Keywords:** piezoelectric-based touch panel, force sensing, detection accuracy, interactive displays, artificial neural network

## Abstract

High detection accuracy in piezoelectric-based force sensing in interactive displays has gained global attention. To achieve this, artificial neural networks (ANN)—successful and widely used machine learning algorithms—have been demonstrated to be potentially powerful tools, providing acceptable location detection accuracy of 95.2% and force level recognition of 93.3% in a previous study. While these values might be acceptable for conventional operations, e.g., opening a folder, they must be boosted for applications where intensive operations are performed. Furthermore, the relatively high computational cost reported prevents the popularity of ANN-based techniques in conventional artificial intelligence (AI) chip-free end-terminals. In this article, an ANN is designed and optimized for piezoelectric-based touch panels in interactive displays for the first time. The presented technique experimentally allows a conventional smart device to work smoothly with a high detection accuracy of above 97% for both location and force level detection with a low computational cost, thereby advancing the user experience, and serviced by piezoelectric-based touch interfaces in displays.

## 1. Introduction

Touch-based interactivity has become a must-have function in smartphones and has created abundant applications in the last decade, improving users human‒machine interactivity (HMI) in a convenient and highly efficient manner. The two-dimensional touch sensing, implemented by capacitive and resistive architectures, has traditionally dominated the market. However, the rapid development of information technology requires more data to be exchanged between the user and the end-terminal nowadays, boosting the popularity of three-dimensional force touch sensing. 

Current force sensing in commercialized smartphones is supported by capacitive (e.g., iPhone 6S) and piezoresistive (e.g., iPhone X) techniques. The former integrates a layer of capacitive sensors into the backlight of the display to measure the distance shift due to the applied force between the cover glass and the backlight. In contrast, the latter utilizes the force-induced resistance change at the inserted electrodes to interpret the force level. To obtain the force sensing functionality without affecting the capacitive touch sensing, both the capacitive and the piezoresistive techniques require additional components, complex readout circuitry, and increased power consumption, all of which are highly undesirable. Hence, the demand for a simpler structured technique for passively detecting force information has been created. To that end, piezoelectric-based techniques have attracted attention for two main reasons: First, they can intrinsically convert mechanical stress into electrical energy, thus achieving force sensing passively. Secondly, they can be employed as the insulating layer of the original capacitive touch panel, obtaining three-dimensional touch sensing at the same time, without adding extra components. 

The piezoelectric force touch panel prototypes have been broadly demonstrated [1,2,3,4,5,6,7]; however, their successful use in commercialized products has not yet been reported. This is due to the issue of non-uniformity of the over-panel force‒voltage responsivity [8,9], which means that the same force strength will give rise to different voltage levels when the touch location varies. To address this issue, artificial neural networks (ANN), which are popular machine learning algorithms, are employed to map voltage levels to touch locations and force levels by training with labeled data. We previously developed an ANN-based technique to interpret the touch locations and force levels, and an acceptable detection accuracy (force detection: 93.3% and location detection: 95.2%) was yielded [9]. Nevertheless, the detection accuracy is still expected to be boosted, since a 93.3% detection accuracy cannot support complex software environments (e.g., action games), which demand intensive operations to be recognized precisely. Furthermore, the complexity of the developed technique strongly limits the technique’s capabilities when implemented in artificial intelligence (AI) chip-free smartphones. Therefore, a modified technique for high detection accuracy in conventional chips is highly desirable, indicating that an optimization of the ANN structure is vital and urgent.

Since no research has yet been reported about the optimization of an ANN for piezoelectric-based force touch panels, in this paper, we investigate the relationship between the ANN hyperparameters (including hidden layer number, node number, cost functions, etc.) and the detection accuracy in terms of location and force level interpretation. We also study the effects of ANN structures (including hidden layer number and node number) on the computational cost, which is directly related to the processing time and power consumption of smart electronic devices and systems. The work presented here reveals the relationship between ANN structure, detection accuracy, and computational cost of the piezoelectric-based force touch panel for the first time, providing design considerations for both the academic and the industrial community. 

This paper is organized as follows: Section 2 briefly reviews the working principle of piezoelectric-based force touch panels and their limitations when being used as interactive interfaces for smartphones; Section 3 explains the methodology; Section 4 demonstrates and discusses the experimental results.

## 2. Literature Review

**Figure 1:**
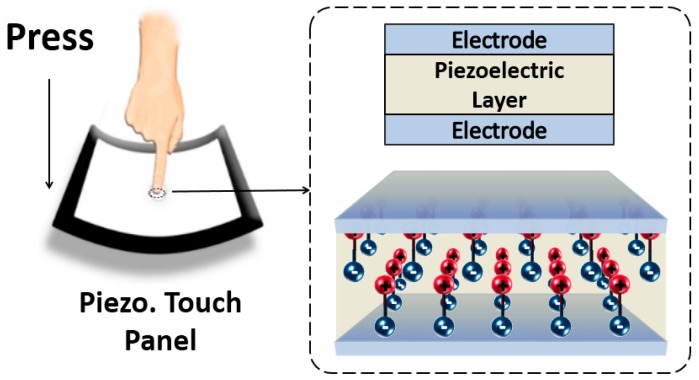
Conceptual depiction of a multi-functional stack-up.

A typical structured piezoelectric force touch panel is shown in Figure 1. It can be observed that three layers are used to construct the touch panel: A thin piezoelectric film layer in the middle for responding to force touch events and two electrode layers, one on top and one underneath the piezoelectric layer, for conveying the force-induced electric signals [10,11,12,13,14,15]. When a force touch is applied at the surface of the piezoelectric touch panel, the polarization of the piezoelectric layer becomes positive or negative according to the direction of the applied force, hence attracting the charges at the electrodes to form an electric potential. The relationship between the applied force and the induced polarization can be expressed as follows [16,17]:

(1) $$P_{i}=d_{ij}\sigma_{jk}\;with\;i,j,k=1,2,3,$$

where *P_i_* is the induced polarization, and *σ_jk_* and *d_ijk_* denote the stress and piezoelectric coefficient, respectively. The coefficients remain the same for direct and inverse piezoelectric effects. The coefficients *d_ijk_* are symmetric in *j* and *k* [10]. Thus, Equation (1) can be simplified as follows:

(2) $$P_{i}=d_{ij}\sigma_{j}\;with\;i=1,2,3\;and\;j=1,2,\ldots ,6$$

From Equations (1) and (2), one can observe that the induced polarization is determined by both the piezoelectric coefficient and the stress vector. The piezoelectric coefficient does not change considerably after fabrication [18]. However, the same force touch can induce different stress vectors when applied at different locations of the touch panel, which is due to the mechanical properties and boundary conditions of the touch panel. As a result, the force‒polarization responsivity is not consistent, which downgrades the detection accuracy [8,9,19].

## 3. Methodology

In this section, the methodology used in this work is described. Details of the experimental setup and data acquisition technique are provided, followed by a description of the pre-processing method and the datasets for training and validation. Finally, the ANN for classification is presented.

### 3.1. Experimental Setup and Data Acquisition

**Figure 2:**
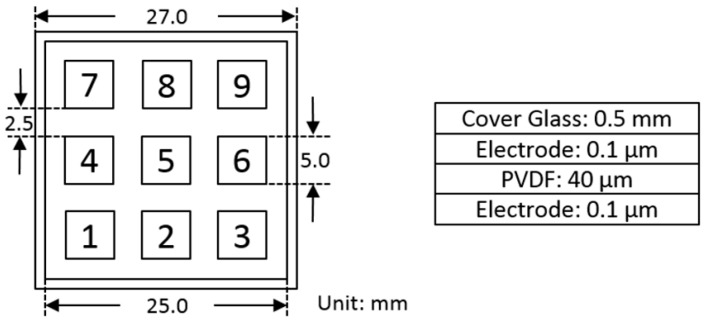
Structure and geometry of the assembled touch panel.

The experiment is carried out with a previously developed multi-layered prototype utilized in Reference [19], consisting of a top cover layer, nine evenly distributed electrodes, a piezoelectric thin film layer, a ground electrode layer, and a bottom cover layer. Figure 2 depicts the topology, together with its geometries.

Four volunteers to perform force touch events are employed for the experiment, and their physical body conditions are listed in Table 1. Each of them is required to tap 1350 times at nine specified locations (as shown in Figure 2) of the touch panel with three different force levels. Hence, a touch set is defined as tapping 50 times per location per force level. Because of the difference in physical body conditions and touch habits of the volunteers, the strength of the three force levels are highly dependent on the individual [19,20,21].

**Figure 3:**
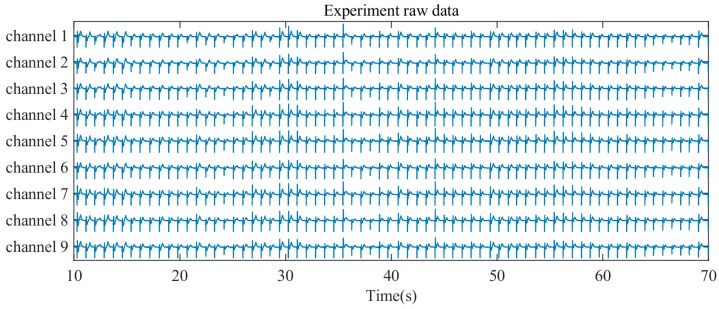
Experimental raw signal data.

The voltages across the nine piezoelectric-based capacitors are continuously monitored. The retrieved raw data for one touch subset are given in Figure 3 as an example, including the raw data from the nine channels; each channel illustrates the relationship between voltage and time. Each peak in the raw data indicates a force touch event. Since the force-induced stress at one location can propagate to other areas, all nine channels produce an output signal when a touch event occurs. However, the amplitudes of the signals are different and depend on the stress distribution on the touch panel. Furthermore, as a complete force touch event consists of “press” and “release” actions, the polarizations of the piezoelectric layer change twice, resulting in positive and negative peaks, as illustrated in Figure 3. The “press” action induces a positive electric signal and is considered to represent the force level of the force touch event.

### 3.2. Pre-Processing and Dataset Preparation

**Figure 4:**
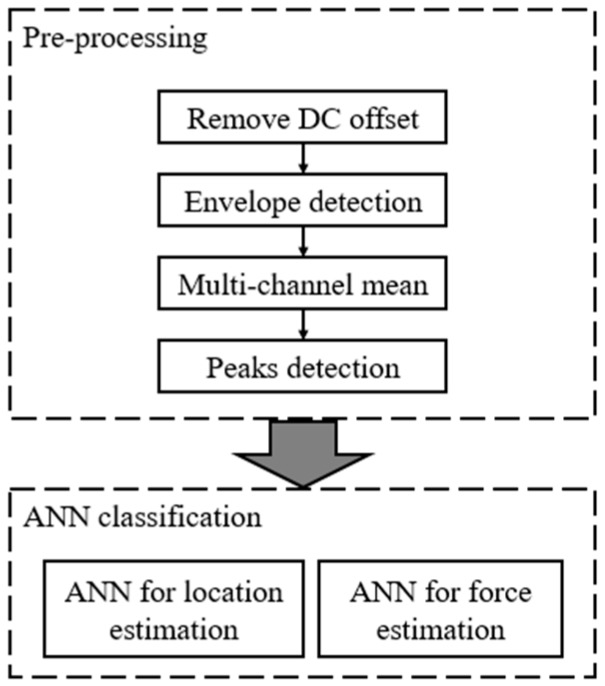
Flow chart for the data pre-processing and classification.

To obtain the information carried in the press-induced electric signals, a pre-processing algorithm has been developed through four steps to extract the voltage peaks from each channel, as shown in Figure 4. First, the direct current (DC) offset of each channel is removed by subtracting the mean voltage of each channel. Second, envelope detection is conducted to each channel by the Hilbert Transform method, as the voltage variations are both positive and negative. Third, we assume that the voltage variation peaks of different channels appear at the same time and calculate the mean value of the nine different channels to suppress random noise. Finally, the voltage peaks are detected by finding the local maximum voltage values. After the voltage peaks for each channel have been acquired, two ANNs are employed to classify locations and force levels. Their details will be given in the next sub-section. 

After pre-processing, nine voltage peaks for each force touch event are obtained and used as features to classify the touch locations and force levels. As mentioned before, 50 touches are performed at each location at the same force amplitude, so 1350 touch signals are obtained in total from each volunteer. Since one touch provides one output data point (including nine peak values from all nine channels), the dataset for a single volunteer consists of 50∙9∙3 output data points. Then, for the training, validation, and testing of the ANN, the dataset is further separated into a training set, a validation set, and a test set. The training set has 40 force touch events for each location and each force level; hence, it has 40∙9∙3 data points, which makes up 80% of the data; the validation and test sets have 10 events each (5∙9∙3 data points, which makes up 10% each).

### 3.3. Pre-Processing Multi-Layers Neural Networks for Classification

Convolution neural networks (CNN) [22,23,24,25,26,27,28,29] and recurrent neural networks (RNN) [30,31,32,33,34,35] are conventionally used to extract features for image and audio applications, and a fully connected network [36,37,38] can be used as an output layer of a CNN or RNN. However, a fully connected network can also be used alone for classification. In our case, the signal features have already been extracted with the help of the pre-processing step explained above, and, thus, we only employed the fully connected network for location and force level classification.

**Figure 5:**
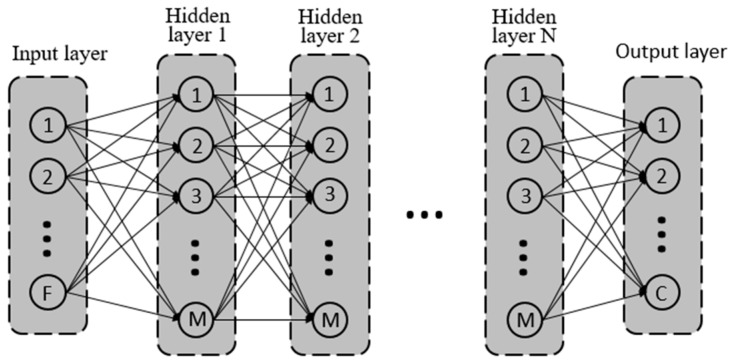
Schematic diagram of a fully connected artificial neural network (ANN). F is the number of features (nine for both force level and location classification); N and M are the hidden layer number and node number; C is the number of object classes (three for force level classification and nine for location classification). Note: Each hidden layer and output layer also have a bias input, which is ignored in this figure.

The fully connected ANN has three kinds of layers with multiple nodes embedded, including an input layer, an output layer, and multiple hidden layers (Figure 5). The number of the input and output layers’ nodes are identical to the number of features and object classes, which are nine and nine for location classification, and nine and three, respectively, for force level classification. Alternatively, the number of hidden layers and their embedded nodes are two independent hyperparameters, which have a significant effect on variance and computational complexity. In general, an increase in the value of these two numbers will have a negative effect on the computational cost; however, ANN becomes more powerful to deal with complex classification scenarios. Thus, the tradeoff between the performance and computational cost must be taken into careful account when designing a fully connected ANN.

The computational time cost includes training time and classification time costs, both of which increase as the layer number and node number increase. The training time cost only occurs once, and that is during the initial training of the ANN. However, the classification time cost occurs every time the user presses the touch panel. Therefore, a high tolerance is usually set for the training time cost, and a low tolerance for the classification time cost. In this study, the training time cost is defined experimentally, while the classification time cost is analyzed theoretically. We implemented our ANNs with different layer numbers and node numbers using Keras. Regarding the training time cost, we trained the ANNs using a laptop (Lenovo ThinkPad X1) with a CPU (Intel(R) Core(TM) i5-3427U @ 1.8GHz), and the time cost of training was recorded. With regard to the classification time cost, it is positively correlated to the number (W) of total weight parameters, which can be calculated by:

(3) $$W=\left(F+C\right)M+\left(N-1\right)M^{2},\;\left(N\geq 1\right),$$

where *F* is the feature number (nine for both force level and location classification), *M* and *N* are the node number and layer number, respectively, and C is the number of object classes. 

The cost function used for training can also greatly affect the performance [39,40,41,42,43,44]. Three commonly used cost functions have been chosen: The mean-squared-error loss function, the categorical cross-entropy loss function, and the binary cross-entropy loss function, and their classification accuracy was compared. 

For the minimization of the loss function during the training process, a stochastic gradient-based optimizer (Adam 2015 [45]) is used to optimize the values of weights and bias of the network, due to its computational efficiency and its low memory requirements [46]. The active function used for the input and hidden layers is ‘ReLU’, since it has high time efficiency [47,48]. In order to avoid overfitting, we make use of L2 regularization with a regularization factor of 0.001, which is selected experimentally [49]. The learning rate is experimentally selected as 0.0005.

## 4. Results and Discussion

**Figure 6:**
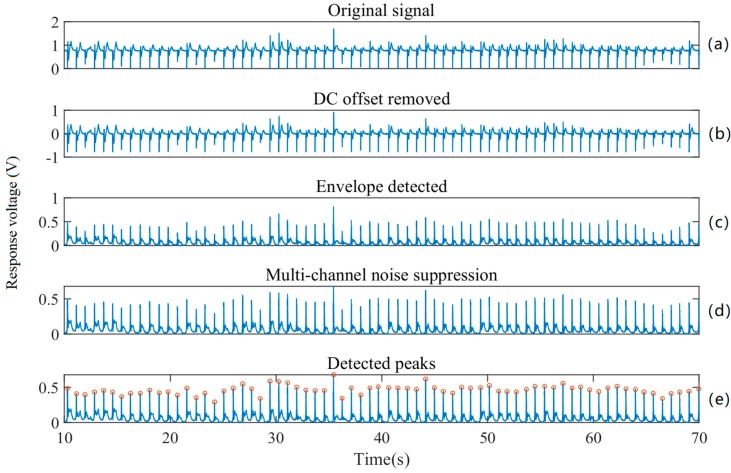
Signal pre-processing results: (**a**) original signal; (**b**) DC offset removed signal; (**c**) signal after envelope detection; (**d**) signal after multi-channel noise suppression; (**e**) detected signal peaks.

**Figure 7:**
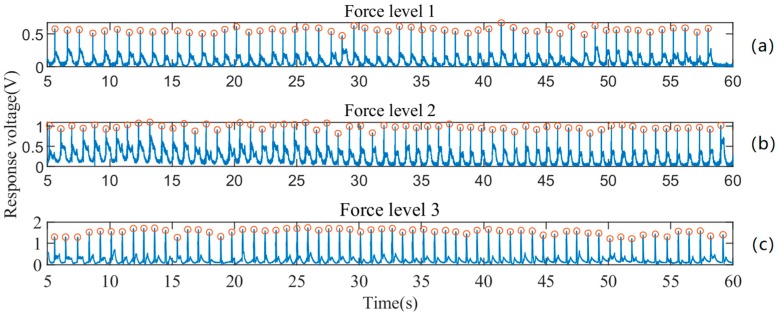
Response voltages at the same location with different force levels; (**a**), (**b**) and (**c**): response voltages of force level 1, 2 and 3.

The pre-processing results after each step are plotted in Figure 6. One can observe that the DC offset at the original output signal is about 0.8 V (Figure 6a). After successfully removing the DC offset, the mean of the signal is close to 0 V (Figure 6b). The envelope detection algorithm is then applied to the offset-free signal, since there are positive and negative components during a force touch event (Figure 6c). Based on the assumption that voltage variation peaks of different channels appear at the same time, the mean value of the nine channels is calculated, as Figure 6d depicts. Finally, the peaks are located and marked by circles in the detected peaks figure. As Figure 6e confirms, the proposed pre-processing method is capable of appropriately detecting the peaks of the signal. Figure 7 shows the response voltages from one volunteer at the same location with different force levels. As shown, our prototype touch panel has different ranges of response voltage for different force levels. 

**Figure 8:**
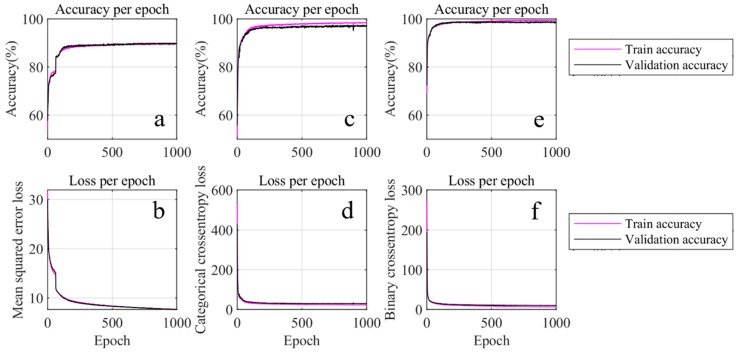
Force level estimation using different loss functions. (**a**,**c**,**e**) are the accuracy during training using the mean-squared error loss, categorical cross-entropy loss, and binary cross-entropy loss, respectively. (**b**,**d**,**f**) are the loss of (**a**,**c**,**e**).

After the pre-processing, the training of the force level classification ANN is performed using three different cost functions, the mean square error cost function, the categorical cross-entropy cost function, and the binary cross-entropy cost function. The corresponding accuracy and loss results are provided in Figure 8. Based on this figure, one can not only observe that the accuracies of both the training set and the validation set are identical, but also that their losses are close to each other. Hence, it is demonstrated that there is no obvious overfitting in this training. Among these three cost functions, the binary cross-entropy cost function provides the best accuracy (98.5%), hence, it is chosen as the cost function for force level classification.

**Figure 9:**
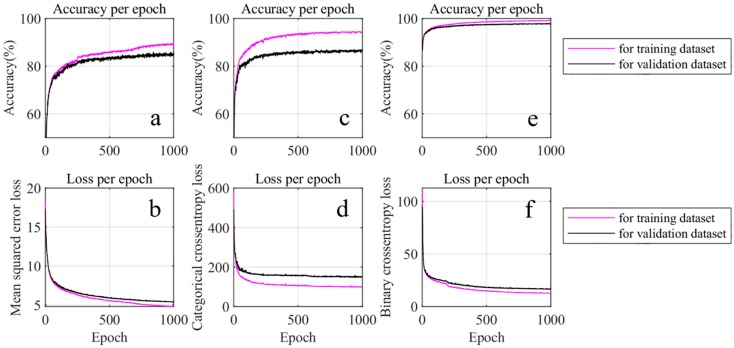
Location estimation using different cost functions. (**a**,**c**,**e**) are accuracy during training using mean-squared error loss, categorical cross-entropy loss, and binary cross-entropy loss, respectively. (**b**,**d**,**f**) are the loss of (**a**,**c**,**e**).

The three cost functions used above are also applied for training of the location classification ANN, and the results are shown in Figure 9. Unlike force level classification, over-fitting appears in two of the three cost functions: The mean-squared error function and categorical cross-entropy cost function. The over-fittings can be seen by observing that the accuracies of the training sets are higher than those of the validation sets. In contrast, the accuracy of the binary cross-entropy cost function for the training set is close to that of the validation set (Figure 9e). In fact, it is 97.8% higher than that of their counterparts. Therefore, the binary cross-entropy cost function is selected for location classification.

**Figure 10:**
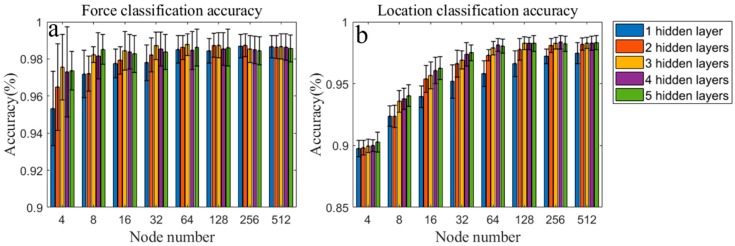
Classification accuracy of ANNs using different layer numbers and node numbers. (**a**): accuracy of force claasification; (**b**): accuracy of location classification.

Figure 10 shows the correlation between the achieved accuracy and the fully connected ANN structure for different layer numbers and node numbers. Figure 10a shows the accuracy of force level classification. For layer numbers 1 to 3 and node numbers 4 to 32, the accuracy increases as the layer number or node number increases. The reason for this is that the ANN structure becomes more complex, hence, it can fit complex classification functions. However, when the layer number becomes larger than 3, and the node number greater than 32, no significant change in the accuracy is observed. Therefore, a layer number of 3 and a node number of 32 are selected for the force level classification in this paper. Figure 10b shows the accuracy of the location classification, which is slightly lower than that of the force level classification. As the layer number increases from 1 to 4 and the node number from 4 to 64, the accuracy also increases. However, for a layer number larger than 3 and a node number larger than 32, the accuracy does not change significantly. Thus, a layer number of 3 and a node number of 64 are chosen for location classification in this paper. Then, the accuracy of the test set is calculated as 97.7% and 97.0% for force and location classification, respectively.

**Figure 11:**
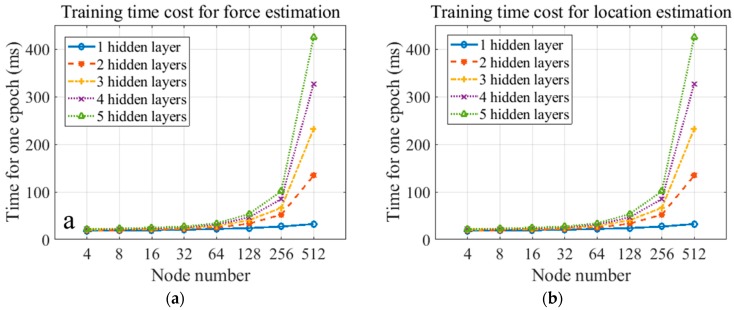
Training time cost for (**a**) force estimation and (**b**) location estimation.

The training time costs for location and force level classification ANNs are given in Figure 11. The time cost remarkably increases when the node number is greater than 64. Also, it rises as the layer number increases. When the ANN has 5 hidden layers and 512 nodes, the maximum training time cost for location and force level classification are 418.2 and 424.8 ms/epoch, respectively.

**Figure 12:**
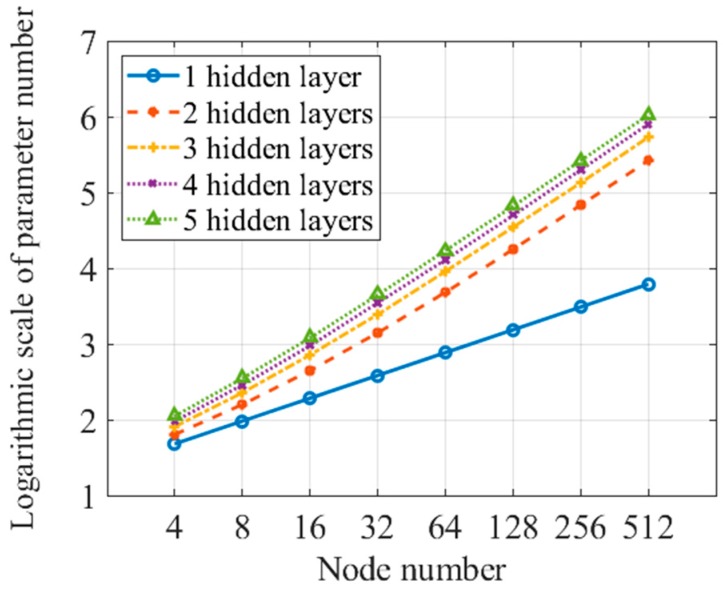
Logarithmic scale of parameter number for force classification ANN.

Figure 12 gives the parameter number for force classification ANN, which is shown on a logarithmic scale, since it grows exponentially. The parameter number for the location classification ANN is a little larger than that of the force level classification ANN, because the output layer node number increases from three–nine. The classification time cost increases as the parameter number increases. Thus, the classification cost also grows exponentially. The location classification time measured for one touch is 0.98 ms for an ANN with 5 hidden layers and 512 nodes for each layer.

## 5. Conclusions

ANNs have been used in the literature to address the inconsistent force‒voltage responsivity issue in piezoelectric-based force touch panels for interactive displays. However, the high computational complexity and the relatively low detection accuracy limit their successful use in conventional consumer products. The work presented in this paper bridges this gap by investigating the relationship between the ANN hyperparameters, detection accuracy, and computational cost. After carefully designing the ANN on the basis of the specific characteristics of the piezoelectric-based touch events, both a high detection accuracy of above 97% and a low computational cost of less than 0.98 ms have been achieved experimentally.

## Figures and Tables

**Table 1:** Main characteristics of the four volunteers.

Volunteer No.	Gender	Height (cm)	Weight (kg)	Handedness
1	Male	173	71	Right
2	Male	182	90.5	Right
3	Female	167	65	Left
4	Female	159	44	Right
